# An increase in red blood cell distribution width from baseline predicts mortality in patients with severe sepsis or septic shock

**DOI:** 10.1186/cc13145

**Published:** 2013-12-09

**Authors:** Chan Ho Kim, Jung Tak Park, Eun Jin Kim, Jae Hyun Han, Ji Suk Han, Jun Yong Choi, Seung Hyeok Han, Tae-Hyun Yoo, Young Sam Kim, Shin-Wook Kang, Hyung Jung Oh

**Affiliations:** 1 Department of Internal Medicine, College of Medicine, Yonsei University, 50 Yonsei-ro, Seodaemoon-Gu, 120-752 Seoul, Korea; 2 Severance Biomedical Science Institute, Brain Korea 21, Yonsei University, 50 Yonsei-ro, Seodaemoon-Gu, 120-752 Seoul, Korea

## Abstract

**Introduction:**

A potential independent association was recently demonstrated between high red blood cell distribution width (RDW) and the risk of all-cause mortality in critically ill patients, although the mechanism underlying this relationship remains unclear. Little is known about the impact changes in RDW may have on survival in critically ill patients. Therefore, we investigated the prognostic significance of changes in RDW during hospital stay in patients with severe sepsis or septic shock.

**Methods:**

We prospectively enrolled 329 patients who were admitted to the emergency department (ED) and received a standardized resuscitation algorithm (early-goal directed therapy) for severe sepsis or septic shock. The relationship between the changes in RDW during the first 72 hours after ED admission and all-cause mortality (28-day and 90-day) were analyzed by categorizing the patients into four groups according to baseline RDW value and ΔRDW_72hr-adm_ (RDW at 72 hours – RDW at baseline).

**Results:**

The 28-day and 90-day mortality rates were 10% and 14.6%, respectively. Patients with increased RDW at baseline and ΔRDW_72hr-adm_ >0.2% exhibited the highest risks of 28-day and 90-day mortality, whereas the patients with normal RDW level at baseline and ΔRDW_72hr-adm_ ≤0.2% (the reference group) had the lowest mortality risks. For 90-day mortality, a significantly higher mortality risk was observed in the patients whose RDW increased within 72 hours of ED admission (normal RDW at baseline and ΔRDW_72hr-adm_ >0.2%), compared to the reference group. These associations remained unaltered even after adjusting for age, sex, Sequential Organ Failure Assessment (SOFA) score, Charlson Comorbidity Index, renal replacement therapy, albumin, hemoglobin, lactate, C-reactive protein and infection sites in multivariable models.

**Conclusions:**

We found that an increase in RDW from baseline during the first 72 hours after hospitalization is significantly associated with adverse clinical outcomes. Therefore, a combination of baseline RDW value and an increase in RDW can be a promising independent prognostic marker in patients with severe sepsis or septic shock.

## Introduction

The red blood cell distribution width (RDW) represents an index of the heterogeneity of the erythrocytes (anisocytosis), which is calculated by dividing the standard deviation of erythrocyte volume by the mean corpuscular volume (MCV) and multiplying by 100 to express the result as a percentage [1]. RDW is widely available to clinicians because it is routinely reported as part of the complete blood count.

For several decades, RDW has been typically used in combination with the MCV to differentiate the cause of underlying anemia in clinical practice [2]. Recently, highly significant associations have been described between RDW value and all-cause, noncardiac, and cardiac mortality in patients with coronary artery disease, acute and chronic heart failure, peripheral artery disease, stroke, pulmonary embolism, and pulmonary artery hypertension [3-8]. Moreover, several studies have reported that RDW shows the predictive value of all-cause mortality in critically ill or intensive care unit (ICU) patients [9-12]. Although it has been postulated that systemic inflammation, malnutrition, and impaired renal function play a significant role in the underlying pathological processes [13], the mechanism of the association between increased RDW and mortality remains unclear.

Until now, most previous studies that have investigated the relationship between RDW and clinical outcomes of various cohorts have used a single RDW measurement at initial presentation, and little is known about the potential impact of changes in RDW from baseline on survival in critically ill patients. However, RDW can be considered as a dynamic variable with rapid changes associated with acute disease states such as acute myocardial infarction and acute decompensated heart failure [14,15]. Thus, we hypothesized that the changes in RDW from baseline can reflect acute disease states and provide more prognostic information than the baseline RDW value alone. Therefore, we investigated whether the change in RDW value between baseline and 72 hours after hospital admission had prognostic value for clinical outcomes in patients with severe sepsis or septic shock.

## Materials and methods

### Patients

Eligible adult patients who were admitted to the emergency department (ED) with severe sepsis and/or septic shock between November 2007 and November 2011 were assessed for possible enrollment according to inclusion and exclusion criteria. Since November 2007, early goal-directed therapy (EGDT) has been implemented in the ICU and in the ED at our institute as part of a quality improvement initiative. If a patient presented with two or more systemic inflammatory response syndrome criteria and a suspicious sign of infection, the patient’s eligibility for EGDT was assessed. One or both of the following triggered initiation of our EGDT protocol: (a) initial systolic blood pressure <90 mmHg, despite a 20 mL/kg intravenous crystalloid fluid challenge; or (b) initial serum lactate level ≥4 mmol/L. The criteria for exclusion included: (a) age <18 years; (b) any contraindication to central venous catheterization; and/or (c) presence of a do-not-resuscitate order.

The study was carried out in accordance with the Declaration of Helsinki and approved by the Institutional Review Board of Yonsei University Health System Clinical Trial Center. We obtained informed written consent from all participants involved in our study.

### Data collection

Baseline characteristics including demographic information and preexisting chronic comorbidities were collected. The Charlson Comorbidity Index (CCI) was used to assess the burden of chronic disease [16,17]. Moreover, both Acute Physiology and Chronic Health Evaluation (APACHE) II score and Sequential Organ Failure Assessment (SOFA) score were determined using the worst values within the initial 24 hours of ED admission for disease severity assessment. SOFA score was calculated by the parameters as follows: PaO_2_/FiO_2_, platelet count, bilirubin, blood pressure and the use of inotropic agent, Glasgow coma score scale, and creatinine or urine output. In addition, RDW, white blood cell (WBC) count, hemoglobin (Hb) level, hematocrit, and MCV were measured at initial presentation and at 72 hours after ED admission, using the Advia 2120 Hematology Analyzer (Siemens Healthcare Diagnostics, Deerfield, IL, USA). RDW is reported as a coefficient of variation (percentage) of red blood cell volume. The normal reference range for RDW in this hospital laboratory is 11.5 to 14.5%.

### Definitions

Sepsis, severe sepsis, and septic shock were defined using the American College of Chest Physicians/Society of Critical Care Medicine consensus conference definitions [18]. Sepsis was defined by two or more of the following conditions as a result of infection: (i) temperature greater than 38°C, (ii) heart rate greater than 90 beats/min, (iii) respiratory rate greater than 20 breaths/min or PaCO_2_ less than 32 mmHg, and (iv) WBC count greater than 12,000 cells/μL or less than 4,000 cells/μL. Severe sepsis was defined as sepsis associated with organ dysfunction, hypoperfusion abnormality, or sepsis-induced hypotension. Hypoperfusion abnormalities included lactic acidosis, oliguria, and acute alteration of mental status. In addition, septic shock was defined as sepsis with hypotension despite adequate fluid resuscitation. Hypotension was defined as a systolic blood pressure of 90 mmHg or less, or a reduction of greater than 40 mmHg from baseline in the absence of other causes of low blood pressure.

Infection sites were categorized as pneumonia, peritonitis, urinary tract infection, exacerbation of chronic obstructive pulmonary disease, catheter-related infection, primary bacteremia (excluding untreated Staphylococcus epidermidis bacteremia), miscellaneous sites (mediastinitis, prostatitis, osteomyelitis, and others), or multiple sites [19]. Effectiveness of antibiotics was assessed based on the microbial culture results, the known susceptibility of the organism to the antimicrobials used, and antimicrobial susceptibility testing [19].

### Statistical analyses

Continuous variables are expressed as means ± standard deviation or median (interquartile ranges), and categorical variables as numbers with percentages. Baseline characteristics of the groups were compared using one-way analysis of variance for continuous variables and the χ^2^ test for categorical variables. In the present study, all patients were followed for 12 months after admission to the ED. We evaluated 28-day and 90-day all-cause mortality as primary endpoints. We also investigated the total length of hospital stay (TLHS) as a secondary endpoint for analysis. The change in RDW between baseline and 72 hours after ED admission (ΔRDW_72hr-adm_) was calculated as RDW at 72 hours – RDW at baseline, and the median value of ΔRDW_72hr-adm_ was 0.2%. Moreover, we categorized patients into four groups according to baseline RDW value and ΔRDW_72hr-adm_ as follows: group 1, patients with RDW levels in the reference range (normal RDW level) at baseline and ΔRDW_72hr-adm_ ≤0.2%; group 2, patients with increased RDW at baseline and ΔRDW_72hr-adm_ ≤0.2%; group 3, patients with normal RDW at baseline and ΔRDW_72hr-adm_ >0.2%; and group 4, patients with increased RDW at baseline and ΔRDW_72hr-adm_ >0.2%. Based on the four groups stratified by baseline RDW value and ΔRDW_72hr-adm_, survival curves were designed using the Kaplan-Meier method, and comparisons were made using the log-rank test. The prognostic value of the changes in RDW on 28-day and 90-day mortality was determined using Cox proportional hazards model, and the results were presented as hazard ratios (HRs) and the 95% confidence intervals (CIs). In multivariate analysis, the Cox models were adjusted for age, sex, SOFA score, CCI, renal replacement therapy (RRT), serum albumin level, Hb, lactate, C-reactive protein (CRP), and infection sites, which were thought to plausibly interact with both RDW and mortality. Furthermore, multivariate linear regression analysis was also performed to determine the factors independently associated with TLSH. All tests were two-sided, and a *P* value of <0.05 was considered statistically significant. Statistical analyses were performed using SPSS for Windows version 19.0 (IBM Corp, Armonk, NY, USA).

## Results

### Baseline characteristics

**Figure 1:**
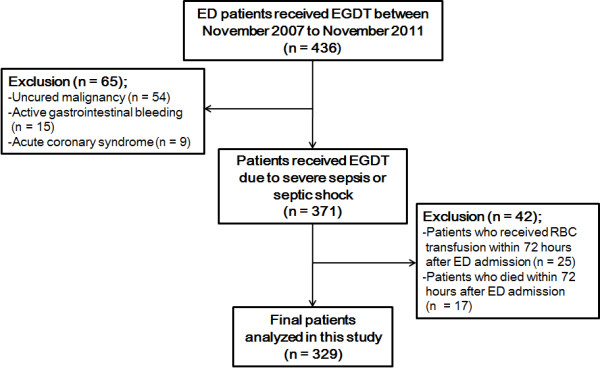
**Flow diagram of study subjects.** From November 2001 to November 2011, 436 patients who received early-goal directed therapy (EGDT) in the emergency department (ED) were assessed for possible enrollment according to inclusion and exclusion criteria, and 329 patients were included in the final analysis.

A total of 436 patients who received EGDT in the ED were initially enrolled in the present study. We excluded 65 patients who were diagnosed with uncured malignancy, active gastrointestinal bleeding, and acute coronary syndrome. Twenty-five patients who received red blood cell (RBC) transfusion within 72 hours after ED admission were also excluded. In the final analysis, we included 329 patients, excepting 17 patients who died within 72 hours after ED admission (Figure 1).

The mean age of the patients was 64.4 ± 15.6 years and 48.9% of patients were male. The mean APACHE II score was 17.5 ± 7.3 and the mean SOFA score was 8.1 ± 2.8. Moreover, RDW levels were from 11.4% to 20.3% (median, 13.5%; mean, 14.0 ± 1.6%) at baseline and from 11.5% to 20.8% (median, 13.8%; mean, 14.1 ± 1.6%) at 72 hours after ED admission, and the main infection sites were urinary tract (24.6%) and lung (24.3%), followed by intra-abdominal cavity (21.9%) (Table 1). The baseline clinical characteristics of each group stratified by baseline RDW value and ΔRDW_72hr-adm_ are presented in Table 1. Compared with the other groups, group 1 exhibited significantly lower age, APACHE II score, SOFA score, and CCI, while baseline Hb, serum albumin, and total cholesterol in group 1 were significantly higher than those in the other groups. Group 4 had the highest lactate level, but the difference did not reach the statistical significance (*P* = 0.065). The proportion of patients who received RRT due to renal failure was highest in group 4.

**Table 1:** **Comparisons of clinical and biochemical variables according to baseline RDW value and ΔRDW**_**72hr-adm**_

**Variables**	**Total (n = 329)**	**Groups based on baseline RDW value and ΔRDW**_**72hr-adm**_^**†**^				***P *** **value**
		**Group 1**	**Group 2**	**Group 3**	**Group 4**	
		**(n = 108)**	**(n = 63)**	**(n = 121)**	**(n = 37)**	
**Demographic data**						
Age (years)	64.4 ± 15.6	59.7 ± 17.9	67.0 ± 12.1	66.3 ± 14.3	67.2 ± 15.7	0.002
Male, n (%)	161 (48.9%)	47 (43.5%)	36 (57.1%)	56 (46.3%)	22 (59.5%)	0.176
MAP (mmHg)	60.0 ± 8.3	60.2 ± 8.7	60.7 ± 7.7	59.7 ± 7.9	59.4 ± 9.8	0.863
BMI (kg/m^2^)	23.0 ± 3.9	23.3 ± 4.1	22.1 ± 4.4	23.3 ± 3.4	22.7 ± 3.9	0.150
APACHE II score	17.5 ± 7.3	14.8 ± 6.4	19.2 ± 17.3	17.3 ± 6.7	23.0 ± 7.6	<0.001
SOFA score	8.1 ± 2.8	7.0 ± 2.5	8.8 ± 2.7	8.1 ± 2.9	9.5 ± 3.0	<0.001
Charlson Comorbidity Index	1.4 ± 1.5	1.1 ± 1.3	2.0 ± 1.7	1.2 ± 1.4	1.5 ± 1.5	0.001
**Biochemical data**						
RDW at baseline (%)	14.0 ± 1.6	13.2 ± 0.7	16.0 ± 1.3	13.0 ± 0.8	15.8 ± 1.1	<0.001
RDW at 72 hours (%)	14.1 ± 1.6	13.0 ± 0.7	15.6 ± 1.3	13.6 ± 0.8	16.5 ± 1.2	<0.001
WBC (x 10^3^/mm^3^)	13.9 ± 9.3	13.0 ± 7.4	15.1 ± 12.0	14.5 ± 9.8	12.2 ± 6.2	0.286
Hemoglobin (g/dL)	12.3 ± 2.2	12.8 ± 2.1	11.5 ± 1.8	12.5 ± 2.0	11.6 ± 2.7	<0.001
Hematocrit (%)	36.6 ± 6.2	37.7 ± 6.1	34.7 ± 5.5	37.0 ± 5.9	35.3 ± 8.0	0.010
CRP (mg/dL)	15.2 ± 11.3	13.1 ± 10.3	14.4 ± 11.8	16.9 ± 11.1	16.7 ± 13.1	0.054
Creatinine (mg/dL)	2.1 ± 1.8	1.7 ± 1.7	2.4 ± 2.1	2.0 ± 1.5	2.6 ± 2.2	0.026
eGFR (mL/min/1.73 m^2^)	54.0 ± 27.7	59.0 ± 26.5	56.9 ± 29.9	49.5 ± 26.5	49.5 ± 29.5	0.045
Albumin (g/dL)	3.3 ± 0.7	3.6 ± 0.6	2.9 ± 0.8	3.4 ± 0.6	2.9 ± 0.8	<0.001
Total cholesterol (mg/dL)	128.7 ± 43.0	138.3 ± 41.9	115.1 ± 44.1	131.4 ± 39.8	114.8 ± 46.3	0.001
Total bilirubin (mg/dL)	1.3 ± 1.5	1.2 ± 1.1	1.3 ± 1.7	1.2 ± 1.1	1.7 ± 2.7	0.224
pH	7.42 ± 0.10	7.43 ± 0.08	7.41 ± 0.12	7.43 ± 0.09	7.39 ± 0.09	0.113
Bicarbonate (mEq/L)	21.0 ± 5.2	21.1 ± 4.9	20.8 ± 5.6	21.3 ± 5.1	20.6 ± 6.1	0.094
Lactate (mmol/L)	3.40 ± 2.80	3.17 ± 2.64	3.77 ± 2.56	3.13 ± 2.93	4.35 ± 3.00	0.065
**Infection site, n (%)**						0.002
Lung (pneumonia)	80 (24.3%)	15 (13.9%)	23 (36.5%)	24 (19.8%)	18 (48.6%)	
Urinary tract	81 (24.6%)	30 (27.8%)	10 (15.9%)	37 (30.6%)	4 (10.8%)	
Intra-abdominal site	72 (21.9%)	29 (26.9%)	10 (15.9%)	26 (21.5%)	7 (18.9%)	
Other	71 (21.6%)	28 (25.9%)	14 (22.2%)	24 (19.8%)	5 (13.5%)	
Multiple sites	25 (7.6%)	6 (5.6%)	6 (9.5%)	10 (8.3%)	3 (8.1%)	
**Acute kidney injury, n (%)**^**‡**^	174 (52.9%)	49 (45.4%)	35 (55.6%)	68 (56.2%)	22 (59.5%)	0.284
**RRT, n (%)**	54 (16.4%)	6 (5.6%)	15 (23.8%)	16 (13.2%)	17 (45.9%)	<0.001
**28-day mortality, n (%)**	33 (10.0%)	2 (1.9%)	11 (17.5%)	8 (6.6%)	12 (32.4%)	<0.001
**90-day mortality, n (%)**	48 (14.6%)	2 (1.9%)	13 (20.6%)	16 (13.2%)	17 (45.9%)	<0.001
**Total length of hospital stay (days)**	19.3 ± 22.8	14.3 ± 11.7	26.9 ± 22.8	16.6 ± 10.8	44.2 ± 64.3	<0.001

Data are expressed as mean (with SD) or n (%). ^†^Group 1 was defined as patients with RDW levels with the reference range (normal RDW level) at baseline and ΔRDW_72hr-adm_ ≤0.2%, group 2 as patients with increased RDW at baseline and ΔRDW_72hr-adm_ ≤0.2%, group 3 as patients with normal RDW at baseline and ΔRDW_72hr-adm_ >0.2%, and group 4 as patients with increased RDW at baseline and ΔRDW_72hr-adm_ >0.2%; ^‡^acute kidney injury was defined as any of the following: (i) increase in serum creatinine by ≥0.3 mg/dL within 48 hours, (ii) increase in serum creatinine to ≥1.5 times baseline, which is known or presumed to have occurred within the prior 7 days, (iii) urine volume <0.5 ml/kg/hour for 6 hours. RDW, red blood cell distribution width; ΔRDW_72hr-adm_, RDW at 72 hours – RDW at baseline; MAP, mean arterial pressure; BMI, body mass index; WBC, white blood cell; CRP, C-reactive protein; eGFR, estimated glomerular filtration rate; RRT, renal replacement therapy.

### An increase in RDW from baseline was significantly associated with mortality

**Figure 2:**
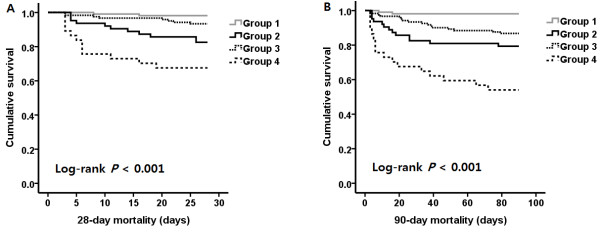
**Kaplan-Meier plots for cumulative 28-day (A) and 90-day (B) survival according to baseline red blood cell distribution width (RDW) value and ΔRDW**_**72hr-adm **_**(ΔRDW**_**72hr-adm**_**, RDW at 72 hours – RDW at baseline).** Group 1 included patients with RDW levels in the reference range (normal RDW level) at baseline and ΔRDW_72hr-adm_ ≤0.2%, group 2 comprised patients with increased RDW at baseline and ΔRDW_72hr-adm_ ≤0.2%, group 3 included patients with normal RDW at baseline and ΔRDW_72hr-adm_ >0.2%, and group 4 was made up of patients with increased RDW at baseline and ΔRDW_72hr-adm_ >0.2% (*P* <0.001 by log-rank test for overall comparison among groups in both 28-day and 90-day cumulative survival plots).

Thirty-three patients (10%) died within 28 days after ED admission, and 48 patients (14.6%) died during the 90-day follow-up. The impacts of the changes in RDW between baseline and 72 hours after ED admission on 28-day and 90-day mortality are shown in Figure 2 and Table 2. Group 1 had the lowest mortality rate during the 28-day and 90-day follow-up compared with the other groups (*P* <0.001, Figure 2).

**Table 2:** **Cox proportional hazards analyses for 28-day and 90-day mortality according to baseline RDW value and ΔRDW**_**72hr-adm**_

**Groups based on baseline RDW value and ΔRDW**_**72hr-adm**_	**Unadjusted model**		**Adjusted model 1**		**Adjusted model 2**	
	**HR (95% CI)**	***P *** **value**	**HR (95% CI)**	***P *** **value**	**HR (95% CI)**	***P *** **value**
**28-day mortality**						
Group 1	1.0	-	1.0	-	1.0	-
Group 2	10.09 (2.24 to 45.52)	0.003	4.57 (0.93 to 22.47)	0.062	4.86 (0.93 to 25.29)	0.060
Group 3	3.64 (0.77 to 17.14)	0.102	1.31 (0.26 to 6.49)	0.743	1.72 (0.34 to 8.69)	0.513
Group 4	21.78 (4.85 to 96.95)	<0.001	7.85 (1.63 to 37.76)	0.010	9.97 (1.99 to 49.91)	0.005
**90-day mortality**						
Group 1	1.0	-	1.0	-	1.0	-
Group 2	12.41 (2.80 to 54.98)	0.001	4.93 (1.06 to 22.94)	0.042	4.72 (1.00 to 22.27)	0.050
Group 3	7.44 (1.71 to 32.34)	0.007	3.85 (0.87 to 17.04)	0.076	4.78 (1.07 to 21.31)	0.040
Group 4	33.41 (7.71 to 144.70)	<0.001	11.66 (2.57 to 52.97)	0.001	13.74 (2.95 to 64.10)	0.001

Unadjusted Model: crude relative risk. Adjusted Model 1: adjusted for age, sex, SOFA score, Charlson Comorbidity Index, renal replacement therapy, albumin, hemoglobin, lactate, and C-reactive protein. Adjusted Model 2: Model 1 plus adjustment for infection site. RDW, red cell distribution width.

In Cox regression analysis of 28-day mortality (Table 2), crude HRs were 10.09 (95% CI, 2.24 to 45.52; *P* = 0.003) in group 2, 3.64 (95% CI, 0.77 to 17.14; *P* = 0.102) in group 3, and 21.78 (95% CI, 4.85 to 96.95; *P* <0.001) in group 4 when group 1 was considered as a reference group (Unadjusted Model). However, after adjusting for age, sex, SOFA score, CCI, RRT, serum albumin, Hb, lactate, and CRP (Model 1), only group 4 showed a significantly higher 28-day mortality risk (HR, 7.85; 95% CI, 1.63 to 37.76; *P* = 0.010). Further adjustment of Model 1 for infection site (Model 2) did not attenuate the significantly higher 28-day mortality risk of group 4 (HR, 9.97; 95% CI, 1.99 to 49.91; *P* = 0.005).

In 90-day mortality (Table 2), crude HRs were 12.41 (95% CI, 2.80 to 54.98; *P* = 0.001) in group 2, 7.44 (95% CI, 1.71 to 32.34; *P* = 0.007) in group 3, and 33.41 (95% CI, 7.71 to 144.70; *P* <0.001) in group 4 when group 1 was considered as a reference group (Unadjusted Model). As in the analysis of 28-day mortality, group 4 also exhibited a significantly higher 90-day mortality risk even after adjusting for age, sex, SOFA score, CCI, RRT, serum albumin, Hb, lactate, CRP, and infection site (HR, 11.66; 95% CI, 2.57 to 52.97; *P* = 0.001 in Model 1, HR, 13.74; 95% CI, 2.95 to 64.10; *P* = 0.001 in Model 2). In addition, the adjusted risk of 90-day mortality was 4.8-fold higher in group 3 (*P* = 0.040 in Model 2), compared to group 1, which was inconsistent with the analysis of 28-day mortality. On the other hand, although the adjusted risk of 90-day mortality was approximately 4.7-fold higher in group 2, it did not reach a statistical significance (*P* = 0.050 in Model 2).

Moreover, the Hosmer-Lemeshow tests were examined to assure the goodness of fit of statistical models and the *P* values for 28-day and 90-day mortality in model 2 were shown to be 0.913 and 0.769, respectively. In addition, the proportional hazards assumption of the four groups, stratified by baseline RDW value and ΔRDW_72hr-adm_, were verified through the parallel lines of the log-minus-log-survival plots.

### An increase in RDW from baseline was significantly associated with total length of hospital stay

Two hundred and seventy-three patients (83.0%) were discharged alive from the hospital, and the median TLHS was 14 days. Group 1 exhibited significantly shorter TLHS than group 4 (14.3 ± 11.7 vs. 44.2 ± 64.3 days; *P* <0.001) (Table 1). Moreover, multivariate linear regression analysis demonstrated that an increase in RDW were significantly associated with TLHS (*β* = 25.45; 95% CI, 14.69 to 36.21; *P* <0.001 in group 4) (Table 3).

**Table 3:** Independent predictors of total length of hospital stay (TLHS) by multivariate linear regression analysis

**Variables**	***β***	**95% confidence interval**	***P *** **value**
**Groups based on baseline RDW value and ΔRDW**_**72hr-adm**_			
Group 1	1.0	-	-
Group 2	8.33	0.04 to 16.62	0.049
Group 3	0.72	−5.24 to 6.67	0.813
Group 4	25.45	14.69 to 36.21	<0.001
**Infection site**			
Urinary tract	1.0	-	-
Intra-abdominal site	1.42	−6.01 to 8.86	0.707
Other	−0.10	−7.91 to 7.72	0.981
Lung (pneumonia)	14.98	6.81 to 23.15	<0.001
Multiple sites	9.43	−3.08 to 21.95	0.139
**Age**	−0.06	−0.23 to 0.107	0.462
**Male (vs. female)**	−1.14	−7.07 to 4.79	0.706
**SOFA score**	1.01	−0.19 to 2.21	0.099
**Charlson Comorbidity Index**	0.08	−1.93 to 2.09	0.937
**RRT (vs. non-RRT)**	2.44	−7.19 to 12.07	0.618
**Albumin (g/dL)**	−2.65	−7.32 to 2.03	0.266
**Hemoglobin (g/dL)**	0.75	−0.65 to 2.14	0.292
**Lactate (mmol/L)**	−0.16	−1.33 to 1.00	0.782
**CRP (mg/dL)**	0.01	−0.02 to 0.03	0.504

RDW, red blood cell distribution width; RRT, renal placement therapy; CRP, C-reactive protein.

## Discussion

The present study is a prospective clinical investigation of the prognostic value of changes in RDW in patients receiving a standardized resuscitation algorithm (EGDT) for severe sepsis or septic shock. The impact of changes in RDW during the first 72 hours after ED admission on all-cause mortality was analyzed by categorizing the patients into four groups according to baseline RDW value and ΔRDW_72hr-adm_ (RDW at 72 hours – RDW at baseline). To the best of our knowledge, this study is the first to report that an increase in RDW has a potential role in predicting mortality in patients with severe sepsis or septic shock. The main finding of the present study is that an increase in RDW from baseline during the first 72 hours after hospitalization can serve as a strong independent predictor of mortality in patients with severe sepsis or septic shock. This significant association between an increase in RDW and mortality remained unaltered even after adjusting for various confounding variables. Group 4 patients, those with increased RDW at baseline and ΔRDW_72hr-adm_ >0.2%, exhibited the highest risks in 28-day and 90-day mortality, while group 1 patients, those with normal RDW level at baseline and ΔRDW_72hr-adm_ ≤0.2%, had the lowest mortality risks. Thus, we speculate that RDW is a dynamic marker of risk in septic patients, and that an increase in RDW during the course of hospitalization can serve as an early indicator of adverse outcomes.

Interestingly, in multivariate analysis for 90-day mortality, a significantly higher mortality risk was observed in group 3 in which RDW increased within 72 hours after ED admission (normal RDW at baseline and ΔRDW_72hr-adm_ >0.2%) compared to group 1, while there was an insignificant difference between group 1 and 3 in 28-day mortality. These findings demonstrate that the relationship between an increase in RDW and mortality may be more meaningful, when the follow-up duration is longer.

Although the mechanism underlying the association between higher RDW and mortality in septic patients is not yet completely understood, several plausible explanations have been suggested in previous studies. Systemic inflammation has been shown to predict progressive illness, cardiovascular mortality and death in ICU patients. Systemic inflammation response impacts bone marrow function and iron metabolism [20,21], and proinflammatory cytokines have been found to inhibit erythropoietin-induced erythrocyte maturation and proliferation, and to downregulate erythropoietin receptor expression, which are associated with RDW increases [22]. Oxidative stress may also be a contributing factor of the association between RDW and mortality. High oxidative stress is present in sepsis through the generation of reactive oxygen species by activated leukocytes [23]. Moreover, it has been proposed that oxidative stress induces an increase in RDW by reducing RBC survival and increasing the release of large premature RBCs into the peripheral circulation [24]. Another explanation may be related to malnutrition. Nutritional markers, including total cholesterol and albumin, are believed to be significantly associated with RDW [13]. In addition, RDW has also been noted to be associated with renal dysfunction, which is known to be closely related with inflammation and malnutrition [13,25]. Recently, Veeranna *et al*. [26] demonstrated the predictive ability of RDW for HbA1c in healthy nondiabetic adults, proposing the possibility of chronic hyperglycemia along with oxidative stress and inflammation as mediators of the association between RDW and adverse outcomes. All things considered, it is reasonable to assume that increased RDW may represent an integrative measure of the multiple harmful pathologic processes, including inflammation, oxidative stress, malnutrition, and renal dysfunction, that simultaneously occur in critical illness. Thus, with respect to the results of this study, we suggest that changes in RDW, especially increasing RDW, reflect the aggravation of inflammation, oxidative stress, nutritional deficiencies, and renal dysfunction, and that the combination of a baseline RDW value and an increase in RDW can be a promising independent prognostic marker in septic patients. However, inflammation, oxidative stress, nutritional deficiencies, and renal dysfunction may not entirely explain why increased RDW is associated with mortality and greater length of hospital stay, because these associations were still significant even after adjusting for total cholesterol, albumin, and SOFA score; therefore further study is needed to determine the mechanism for the association between RDW values and mortality.

There are several limitations to our study. First, we arbitrarily determined 72 hours after ED admission as the second RDW measurement and defined an increase in RDW as ΔRDW_72hr-adm_ >0.2%. Whether changes in RDW during the first 72 hours could represent the pathophysiologic changes and therapeutic responses in critically ill patients is not clear. Ku *et al*. [27] suggested that 72-hour RDW after the onset of bacteremia could be a predictor of all-cause mortality in patients with Gram-negative bacteremia. Therefore, based on this previous study, we investigated the clinical outcomes of the patients with severe and/or septic shock stratified by the changes in RDW values between baseline and 72 hours after admission. Second, we did not investigate the use of erythropoietin, iron or vitamin B_12_ deficiency, and reticulocyte count, which could have affected RDW values and, thus, might have limited the interpretation of study results. Third, the low number of patients in group 4 might introduce significant interference in the use of statistical models with numerous covariates. We examined two aspects of our models to assure the goodness of fit of our statistical models. For one thing, we conducted multivariate logistic regression analyses with the covariates used in Adjusted Model 2 and examined the results of the Hosmer-Lemeshow tests. Therefore, we confirmed that the *P* values for 28-day and 90-day mortality were 0.913 and 0.769, respectively, which demonstrated that these statistical analyses with numerous variables were acceptable. Next, we examined the proportional hazards assumption of the four groups, stratified by baseline RDW value and ΔRDW_72hr-adm_, through log-minus-log-survival plot, and the parallel lines of the log-minus-log function were verified. Taken together, we suggested that these statistical analyses were tolerable, even though the number of patients in group 4 was relatively small. Furthermore, we analyzed the data with a simplified grouping in the same frame of statistical models. Patients were categorized into four groups based on whether RDW level was within the normal range or increased at baseline and at 72 hours. Group 1 was defined as patients with normal RDW at both time points, group 2 as patients with increased RDW at baseline and normal RDW at 72 hours, group 3 as patients with normal RDW and increased RDW at 72 hours, and group 4 as patients with increased RDW at both time points. In the results, we found that an increase in RDW from baseline was significantly associated with mortality. Moreover, group 4 in this subanalysis exhibited the highest 28-day and 90-day mortality rates similar to our outcomes (See Additional files 1 and 2). Finally, the sample size and the number of events were not large enough to establish statistical significance of increased risk among categorized groups. Therefore, a larger multicenter study with repeated RDW measurements is necessary to further clarify the predictive value of changes in RDW.

## Conclusions

We found that an increase in RDW from baseline during the first 72 hours after hospitalization is significantly associated with adverse clinical outcomes. Therefore, a combination of baseline RDW value and an increase in RDW can be a promising independent prognostic marker for mortality in patients with severe sepsis or septic shock. Although further research is required to determine the precise mechanisms underlying the association between RDW and mortality, this study provides support for future investigations considering changes of RDW and the associated stratification of critically ill patients at risk for mortality.

## Key messages

• The changes in RDW from baseline can reflect acute disease states and provide more prognostic information than the baseline RDW value alone.

• In this single-center prospective observational study including 329 patients, an increase in RDW from baseline during the first 72 hours after hospitalization is significantly associated with adverse clinical outcomes in patients with severe sepsis and septic shock.

• A combination of baseline RDW value and changes in RDW can be a promising independent prognostic marker for mortality in patients with severe sepsis or septic shock.

## Abbreviations

APACHE: Acute physiology and chronic health evaluation; CCI: Charlson Comorbidity Index; CI: Confidence interval; CRP: C-reactive protein; ED: Emergency department; EGDT: Early-goal directed therapy; Hb: Hemoglobin; HR: Hazard ratio; ICU: Intensive care unit; MCV: Mean corpuscular volume; RBC: Red blood cell; RDW: Red blood cell distribution width; RRT: Renal replacement therapy; SOFA: Sequential Organ Failure Assessment; TLHS: Total length of hospital stay; WBC: White blood cell.

## Competing interests

The authors declare they have no competing interests.

## Authors’ contributions

CHK performed the data review/collection, the statistical analyses and developed the initial draft of the manuscript. EJK, JHH, and JSH were involved in the collection and assembly of data. JYC and YSK organized the data collection. JTP, SHH, and HJO revised the draft of manuscript and assisted in data analysis. HJO, THY, and SWK contributed to the study design and coordination of the study. All authors read and approved the final manuscript.

## Supplementary Material

Additional file 1: Table S1 Comparisons of clinical and biochemical variables according to red blood cell distribution width (RDW) level at baseline and at 72 hours. **Table S2:** Cox proportional hazards analyses for 28-day and 90-day mortality according to the changes in RDW. Click here for file

Additional file 2: Figure S1 Kaplan-Meier plots for cumulative 28-day **(A)** and 90-day **(B)** survival according to the changes in red blood cell distribution width (RDW) during the first 72 hours. Click here for file
