# Supplementary material for: An increase in red blood cell distribution width from baseline predicts mortality in patients with severe sepsis or septic shock
Source: Crit Care. 2013 Dec 9;17(6):R282. doi: 10.1186/cc13145 (PMC4056357; doi:10.1186/cc13145)
Supplement: Additional file 1: Table S1 — Comparisons of clinical and biochemical variables according to red blood cell distribution width (RDW) level at baseline and at 72 hours. Table S2: Cox proportional hazards analyses for 28-day and 90-day mortality according to the changes in RDW. [file cc13145-S1.pdf]

Supplementary Table 1. Comparisons of clinical and biochemical variables according to RDW level at baseline and 72-hour

Variables	Total (n = 329)	Groups based on RDW levels at baseline and 72-hour†				*P*-value
		Group 1 (n = 208)	Group 2 (n = 11)	Group 3 (n = 21)	Group 4 (n = 89)	
**Demographic data**						
Age (years)	64.4±15.6	62.8±16.3	68.3±11.9	66.3±17.2	66.9±13.7	0.147
Male, n (%)	161 (48.9%)	93 (44.7%)	6 (54.5%)	10 (47.6%)	52 (58.4%)	0.183
MAP (mmHg)	60.0±8.3	60.0±8.6	59.5±4.8	59.1±4.8	60.3±8.9	0.943
BMI (kg/m²)	23.0±3.9	23.4±3.8	22.3±2.2	23.0±2.9	22.3±4.4	0.183
APACHE II score	17.5±7.3	15.8±6.6	14.7±6.8	19.5±6.8	21.3±7.5	<0.001
SOFA score	8.1±2.8	7.5±2.6	8.9±2.5	9.1±3.3	9.0±2.9	<0.001
Charlson comorbidity index	1.4±1.5	1.1±1.3	1.6±1.6	1.6±1.7	1.9±1.6	0.001
**Biochemical data**						
RDW at baseline (%)	14.0±1.6	13.0±0.7	15.0±0.4	14.2±0.3	16.1±1.2	<0.001
RDW at 72-hour (%)	14.1±1.6	13.2±0.7	14.1±0.4	14.8±0.2	16.1±1.3	<0.001
WBC (x 10³/mm³)	13.9±9.3	13.8±8.8	11.9±5.5	13.9±9.2	14.3±10.7	0.879
Hemoglobin (g/dL)	12.4±2.1	12.9±2.0	11.8±2.2	11.5±1.8	11.7±2.0	<0.001
Hematocrit (%)	37.1±5.9	37.9±5.7	35.2±5.8	34.6±6.0	35.6±6.3	0.006
CRP (mg/dL)	15.2±11.3	14.9±11.0	8.3±9.0	17.2±9.6	16.2±12.4	0.140
Creatinine (mg/dL)	2.1±1.8	1.9±1.5	1.8±1.8	2.3±2.1	2.5±2.2	0.020
eGFR (mL/min/1.73m²)	54.0±27.7	54.1±26.9	63.5±21.6	52.7±27.2	52.9±30.6	0.719
Albumin (g/dL)	3.3±0.7	3.5±0.6	3.2±0.7	3.0±0.7	2.9±0.8	<0.001
Total cholesterol (mg/dL)	128.7±43.0	135.2±40.2	135.6±64.9	128.9±48.1	112.4±41.3	<0.001
Total bilirubin (mg/dL)	1.3±1.5	1.1±1.1	2.0±3.4	1.5±1.2	1.4±1.9	0.168
pH	7.42±0.10	7.43±0.07	7.36±0.22	7.40±0.16	7.41±0.09	0.075
Bicarbonate (mEq/L)	21.0±5.0	21.1±4.9	21.3±5.1	20.8±5.6	20.6±6.1	0.094
Lactate (mmol/L)	3.40±2.80	2.96±2.42	4.12±3.38	5.00±4.91	3.96±2.66	0.001
**Infection sites, n (%)**						0.001
Pneumonia	80 (24.3%)	34 (16.3%)	6 (54.5%)	5 (23.8%)	35 (39.3%)	
Urinary tract	81 (24.6%)	65 (31.3%)	1 (9.1%)	2 (9.5%)	13 (14.6%)	
Intra-abdominal sites	72 (21.9%)	50 (24.0%)	3 (27.3%)	5 (23.8%)	14 (15.7%)	
Other	71 (21.6%)	45 (21.6%)	1 (9.1%)	7 (33.3%)	18(20.2%)	
Multiple sites	25 (7.6%)	14 (6.7%)	-	2 (9.5%)	9 (10.1%)	
**Acute kidney injury, n (%)‡**	174 (52.9%)	103 (49.5%)	4 (36.4%)	14 (66.7%)	53 (59.6%)	0.149
**RRT, n (%)**	54 (16.4%)	16 (7.7%)	3 (27.3%)	6 (28.6%)	29 (32.6%)	<0.001
**28-day mortality, n (%)**	33 (10%)	7 (3.4%)	2 (18.2%)	3 (14.3%)	21 (23.6%)	<0.001
**90-day mortality, n (%)**	48 (14.6%)	12 (5.8%)	2 (18.2%)	6 (28.6%)	28 (31.5%)	<0.001
**Total length of hospital stay (days)**	19.3±22.8	15.1±11.3	20.0±22.6	10.6±2.7	33.8±41.9	<0.001

Data are expressed as mean (with SD) or n (%).

RDW, red cell distribution width; $\Delta RDW_{72hr\text{-}adm}$, RDW at 72-hour – RDW at baseline; MAP, mean arterial pressure; BMI, body mass index; WBC, white blood cell; CRP, C-reactive protein; eGFR, estimated glomerular filtration rate; RRT, renal replacement therapy.

†The group 1 was defined as patients with normal RDW at both time points, the group 2 as patients with increased RDW at baseline and normal RDW at 72-hour, the group 3 as patients with normal RDW and increased RDW at 72-hour, and the group 4 as patients with increased RDW at both time points.

‡Acute kidney injury was defined as any of the following: (i) increase in serum creatinine by ≥ 0.3 mg/dL within 48 hours, (ii)

increase in serum creatinine to ≥ 1.5 times baseline which is known or presumed to have occurred within the prior 7days, (iii) urine volume < 0.5ml/kg/hour for 6hours.

Supplementary Table 2. Cox proportional hazards analyses for 28-day and 90-day mortality according to the changes in RDW

Groups based on RDW levels at baseline and 72-hour	Unadjusted Model		Adjusted Model 1		Adjusted Model 2	
	HR (95% CI)	*P*-value	HR (95% CI)	*P*-value	HR (95% CI)	*P*-value
**28-day mortality**						
Group 1	1.0	-	1.0	-	1.0	-
Group 2	5.72 (1.19-27.56)	0.030	3.59 (0.63-20.50)	0.150	3.55 (0.57-22.17)	0.176
Group 3	4.60 (1.19-17.81)	0.027	1.41 (0.20-9.88)	0.724	1.18 (0.15-9.10)	0.871
Group 4	7.93 (3.37-18.67)	<0.001	5.70 (2.03-15.99)	0.001	5.38 (1.84-15.68)	0.002
**90-day mortality**						
Group 1	1.0	-	1.0	-	1.0	-
Group 2	3.45 (0.77-15.41)	0.105	1.81 (0.37-8.81)	0.461	1.51 (0.29-7.77)	0.621
Group 3	5.60 (2.10-14.91)	0.001	2.42 (0.73-8.10)	0.151	2.45 (0.70-8.57)	0.159
Group 4	6.47 (3.29-12.74)	<0.001	3.65 (1.63-8.15)	0.002	3.31 (1.45-7.56)	0.005

RDW, red cell distribution width.

Unadjusted Model: crude relative risk

Adjusted Model 1: adjusted for age, sex, SOFA score, Charlson Comorbidity Index, renal replacement therapy, albumin, hemoglobin, lactate, and C-reactive protein

Adjusted Model 2: Model 1 plus adjustment for infection site

Supplementary Figure 1. Kaplan-Meier plots for cumulative 28-day (A) and 90-day (B) survival according to the changes in RDW during first 72 hours.

Group 1 was defined as patients with normal RDW at both time points, group 2 as patients with increased RDW at baseline and normal RDW at 72-hour, group 3 as patients with normal RDW and increased RDW at 72-hour, and group 4 as patients with increased RDW at both time points. (P < 0.001 by log-rank test for overall comparison among groups in both 28-day and 90-day cumulative survival plots)
