# Supplementary figures and images for: An increase in red blood cell distribution width from baseline predicts mortality in patients with severe sepsis or septic shock
Source: Crit Care. 2013 Dec 9;17(6):R282. doi: 10.1186/cc13145 (PMC4056357; doi:10.1186/cc13145)

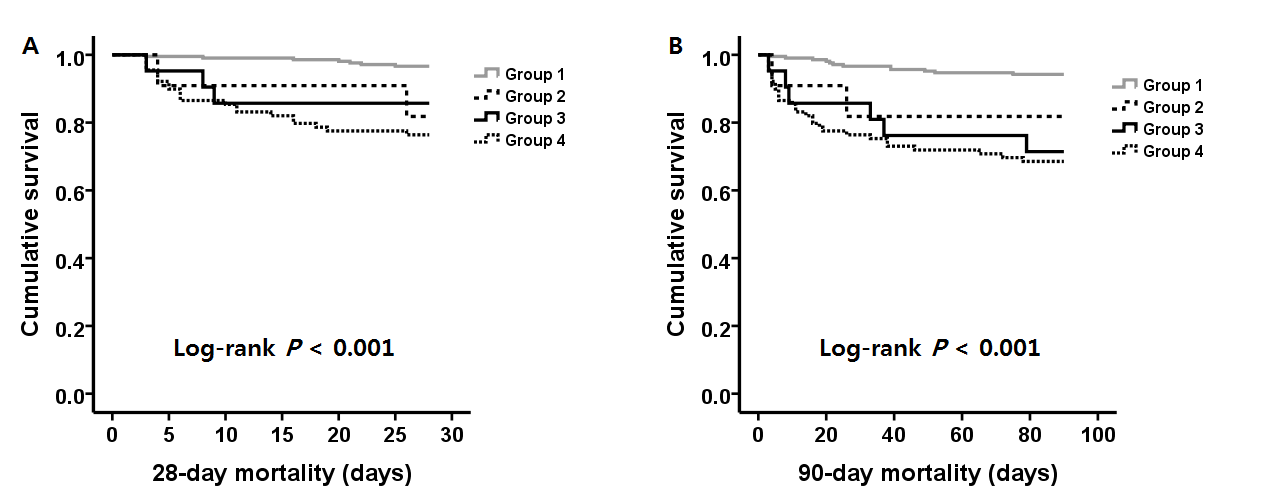

Supplement: Additional file 2: Figure S1 — Kaplan-Meier plots for cumulative 28-day (A) and 90-day (B) survival according to the changes in red blood cell distribution width (RDW) during the first 72 hours. [file cc13145-S2.tiff]
